# Supplementary material for: Dynamics and stability of running on rough terrains
Source: R Soc Open Sci. 2019 Mar 13;6(3):181729. doi: 10.1098/rsos.181729 (PMC6458357; doi:10.1098/rsos.181729)
Supplement: Supplementary notes [file rsos181729supp1.pdf]

Supplementary material
Dynamics and stability of running on rough terrains

Nihav Dhawale[1,2], Shreyas Mandre[3], and Madhusudhan Venkadesan*[1]

[1]Department of Mechanical Engineering and Materials Science, Yale University, New Haven, CT 06520
[2]National Centre for Biological Sciences–Tata Institute of Fundamental Research, Bangalore, Karnataka 560065
[3]School of Engineering, Brown University, Providence, RI 02912

Contents

S1 Methods **S2**
S1.1 Simulation methods S2
S1.2 Parameter values for a human-like runner S2
S1.3 Terrain model S2
S1.4 Calculating ground contact point S2

S2 Convergence of the Monte Carlo simulations **S3**

S3 Model Details **S3**
S3.1 Passive collision S3
S3.2 Relationship between ϵ_{t} and ϵ_{tc} S4
S3.3 Open-loop push-off strategies S6

S4 Open-loop runners on rough terrain **S6**
S4.1 The open-loop passive collision S6
S4.2 Additive noise in open-loop strategies S7

S5 Anticipatory runners on rough terrain **S7**
S5.1 Forward collision parameter $\hat{\epsilon}_{\mathrm{t}}$ as a function of ϵ_{tc} S9

S6 Linear stability analysis: Jordan decomposition of $\boldsymbol{T}_{\mathrm{ol}}$ and $\boldsymbol{T}_{\mathrm{an}}$ **S9**

S7 Scaling analysis of orientation failures **S11**

S8 Steps to failure statistics **S13**

S9 Effect of changing runner size relative to terrain grid spacing **S15**

S10 Monte Carlo simulations with a *laboratory-fixed* push-off **S16**
S10.1 Open-loop runners S17
S10.2 Anticipatory runners S18
S10.3 Scaling analysis of mean steps taken S19

*Address correspondence to mv@classicalmechanic.net

S1 Methods

S1.1 Simulation methods

All simulations were performed using custom-written C programs. Parameter values given in table 1 are used for simulations of the human-like runner. These values are chosen for the purpose of illustration, however our qualitative results are not sensitive to these values, and the scaling analysis in section 5 of the main text addresses the generalization of these numerical results to runners and terrains with varying parameter values.

S1.2 Parameter values for a human-like runner

The rationale for choosing the human-like parameter values is as follows. The moment of inertia value we use is derived from estimates made by Erdmann [1], who find that moment of inertia about the center of mass in the sagittal plane is ≈ 13 kg.m^2 for a 75 kg human. The value for $\epsilon_\mathrm{n} = 0.63$ corresponds to $\approx 40\%$ elastic energy stored and released over one gait cycle, similar to estimates by Alexander et al. [2], Cavagna and Kaneko [3], Cavagna et al. [4]. The orientation bound ϕ_tol is equal to $\pi/6$ as it is approximately half the angle between the legs during double stance in walking. Forward speed at take-off $v_{x0} = 0.96$ corresponds to 3 m/s for a leg length of 1 m and vertical speed at take-off $v_{y0} = 0.26$ corresponds to ≈ 0.8 m/s [5]. Distributions had nearly converged by an ensemble size of 10^4, hence we simulate for 10^5 instances (section S2).

S1.3 Terrain model

The terrain is modeled as piecewise linear. This is achieved by first defining a one-dimensional grid with fixed grid spacing λ. Interpolating heights between the grid points k, located at x_k to intermediate points (x_t, y_t) in the kth terrain patch, yields a piecewise linear, continuous terrain profile, where terrain slope m_k is discontinuous at the grid points,

$$\text{patch } k:\ y_t = m_k x_t + c_k, \tag{S1a}$$

$$\text{where } x_t \in [x_k, x_{k+1}], \tag{S1b}$$

$$\text{continuity condition: } m_k x_{k+1} + c_k = m_{k+1} x_{k+1} + c_{k+1}, \tag{S1c}$$

where m_k and c_k are constants within a patch. Terrain heights at all grid points are distributed according to $\sim \mathcal{U}(-0.03, 0.03)$ (main text table 1). Our choice of the uniform distribution $\mathcal{U}$ is to improve simulation speed, even though beta distributions described in main text figure 2e most closely matched artificial terrain used in experiments [5, 6]. The range of heights $h \in [-0.03, 0.03]$ and grid spacing $\lambda = 0.1$ was chosen to match the artificially constructed rough terrains [5, 6].

Step-like terrains with no slope distributions were simulated by picking a height from the probability distribution prior to landing. If the chosen landing height was above the apex height of any portion of the runner, we chose another landing height from the distribution. The probability of this re-sampling occurring is $\sim 10^{-4}$.

S1.4 Calculating ground contact point

The aerial phase ends when the runner collides with the ground. The landing position is determined by solving for the unknown intersection point x_t of the runner's aerial phase trajectory $(x_G(t), y_G(t))$

with the condition for tangential contact between runner and ground,

$$\text{parabolic flight: } y_{\mathrm{G}} = b_0 + b_1 x_{\mathrm{G}} + b_2 x_{\mathrm{G}}^2, \tag{S2a}$$

$$\text{touchdown: } y_{\mathrm{G}} = y_t + \frac{1}{\sqrt{1+m_k^2}},\ x_{\mathrm{G}} = x_t - \frac{m_k}{\sqrt{1+m_k^2}}, \tag{S2b}$$

where b_0, b_1, b_2 are constants that define the aerial phase trajectory. Equations (S1)-(S2) solved simultaneously yield a quadratic equation in x_t,

$$Ax_t^2 + Bx_t + C = 0, \tag{S3a}$$

$$\text{where } A = b_2, \tag{S3b}$$

$$B = b1 - 2b_2 \frac{m_k}{\sqrt{1+m_k^2}} - m_k, \tag{S3c}$$

$$C = b_0 - \frac{b_1 m_k - 1}{\sqrt{1+m_k^2}} + b_2 \frac{m_k^2}{1+m_k^2} - c_k. \tag{S3d}$$

The larger of the two roots of this quadratic is the true landing point x_{P}, if the roots are real and the larger of the two roots is greater than x_k. The other real root is always less than x_k and corresponds to the intersection of the aerial phase trajectory with the terrain patch closer to the take-off point. On flat terrain, the smaller root is the location of the take-off point. Having solved for x_{P}, the position of the center of mass at landing is determined using equation (S2b).

However, if the runner lands on a grid point, the position of the center of mass would be indeterminate as the grid point x_k is associated with two slopes, m_k and m_{k+1}. In fact, we detect a corner collision if the larger root of equation (S3a) is less than x_k, or if the roots are complex. Thus, we now know the position of contact point P, $x_{\mathrm{P}} = x_k$, but cannot determine (x_G, y_G) at contact using equation (S2b), since x_k is associated with slopes m_k and m_{k+1}. We regularize the slope at the point x_k by accounting for the aerial phase trajectory. Substituting $x_t = x_k$ in equation (S3a), we write equations (S3) as a quartic polynomial in unknown m_k. We numerically find all the roots using the Jenkins-Traub algorithm [7] and pick the real root that corresponds to first contact between the ground and runner, i.e. when the parabolic trajectory describing the aerial phase is above the ground.

S2 Convergence of the Monte Carlo simulations

We ran Monte Carlo simulations with different ensemble sizes to test for convergence of the steps to failure distributions. We define the criterion for convergence as the first three moments of the distribution remaining unchanged to one significant decimal place as a function of ensemble size. This criteria is met with an ensemble size of 10^4 (figure S1). Hence we perform Monte Carlo simulations in the main text with an ensemble size of at least 10^5.

S3 Model Details

S3.1 Passive collision

A runner with center of mass velocity $\boldsymbol{v}_{\mathrm{G}}^-$ and angular velocity ω^- collides with a terrain patch angled at θ with respect to the horizontal. By simultaneously solving equations describing the collision at the contact point P (main text equation (1a)), and using kinematic constraints associated

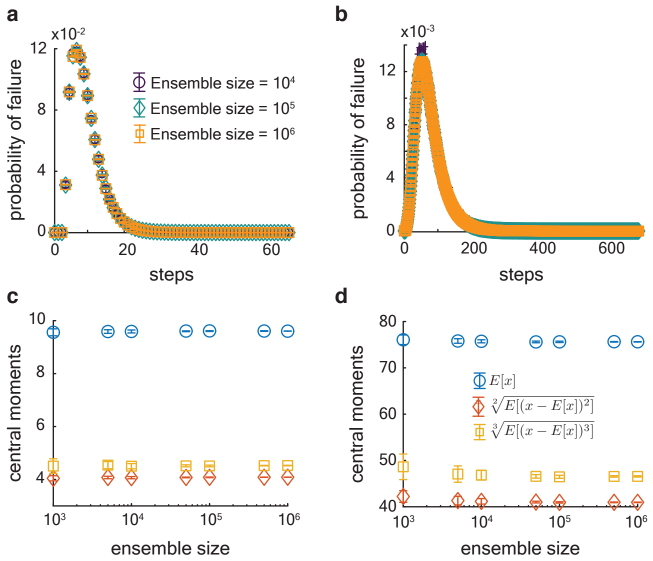

Figure S1: Monte Carlo simulations of open-loop and anticipatory runners with human-like parameter values and varying ensemble sizes. Ten simulations for each ensemble size were performed. Open markers represent the mean value, with error bars showing the standard deviation over the 10 runs. Steps to failure distributions for the (**a**) Open-loop runner and (**b**) Anticipatory runner. The y-axis of the plots is the joint probability p(falling at step number i,reaching step number i). The first three central moments as a function of ensemble size for the (**c**) Open-loop runner and (**d**) Anticipatory runner are stationary for ensemble sizes greater than 10^4.

with a rigid body, the linear velocity $\boldsymbol{v}^c_{\mathrm{G}}$ and angular velocity ω^c of the runner after the collision are computed as,

$$\text{collision law: } v^c_{\mathrm{P,t}} = \epsilon_{\mathrm{t}} v^-_{\mathrm{P,t}}, \tag{S4a}$$

$$v^c_{\mathrm{P,n}} = -\epsilon_{\mathrm{n}} v^-_{\mathrm{P,n}}, \tag{S4b}$$

$$H^c_{/\mathrm{P}} - H^-_{/\mathrm{P}} = 0, \tag{S4c}$$

$$\text{kinematic constraint: } \boldsymbol{v}_{\mathrm{G}} = \boldsymbol{v}_{\mathrm{P}} - \omega \begin{pmatrix} \cos\theta \\ -\sin\theta \end{pmatrix}, \tag{S4d}$$

$$\implies \boldsymbol{v}^c_{\mathrm{G}} = \begin{pmatrix} \frac{(v^-_{\mathrm{G,x}} \cos 2\theta + v^-_{\mathrm{G,y}} \sin 2\theta)A + v^-_{\mathrm{G,x}} B + C\omega^- \cos\theta}{2(I_{/\mathrm{G}}+1)} \\ \frac{(v^-_{\mathrm{G,x}} \sin 2\theta - v^-_{\mathrm{G,y}} \cos 2\theta)A + v^-_{\mathrm{G,y}} B + C\omega^- \sin\theta}{2(I_{/\mathrm{G}}+1)} \end{pmatrix}, \tag{S4e}$$

$$\text{and } \omega^c = \frac{(\epsilon_{\mathrm{t}} + I_{/\mathrm{G}})\omega^- - (1-\epsilon_{\mathrm{t}}) v^-_{\mathrm{G,t}}}{1 + I_{/\mathrm{G}}}, \tag{S4f}$$

$$\text{where } A = I_{/\mathrm{G}}(\epsilon_{\mathrm{t}} + \epsilon_{\mathrm{n}}) + \epsilon_{\mathrm{n}} + 1,\ B = \epsilon_{\mathrm{t}} I_{/\mathrm{G}} - (I_{/\mathrm{G}} + 1)\epsilon_{\mathrm{n}} + 1,\ C = 2(\epsilon_{\mathrm{t}} - 1) I_{/\mathrm{G}}, \tag{S4g}$$

$$\text{and } v_{\mathrm{G,t}} = v_{\mathrm{G,x}} \cos\theta + v_{\mathrm{G,y}} \sin\theta. \tag{S4h}$$

S3.2 Relationship between ϵ_{t} and ϵ_{tc}

Based on a detailed consideration of how an animal may use its foot speed to control the tangential collision, we arrive at equation (2) in the main text. The velocity of the foot and center of mass are not constrained to be the same for a running animal. For example, the animal may retract the leg to

vary the foot's speed independent of the center of mass speed. We make the simplifying assumption that the foot comes to rest, or nearly so, upon colliding with the ground. In an animal, there is an additional degree of control corresponding to modulating the transmission of the collision between the foot and center of mass through changes in the leg posture and muscle co-contraction. We do not include this additional mode of controlling collisions in our consideration.

The use of the foot enables the runner to reduce the effective collision independent of the body's overall linear and angular momentum. Thus the tangential coefficient of restitution ϵ_{t} parametrizes the loss of speed of the foot relative to a point on a rigid runner that does not have the ability to modulate its foot speed. The foot speed of such a rigid runner is that of the contact point P in the rigid running model (main text figure 1b), one that translates with the center of mass and is also affected by any spin that the body may have. The tangential coefficient of restitution ϵ_{t} at the moment of collision is therefore given by,

$$\epsilon_{\mathrm{t}} = \frac{v^{-}_{\mathrm{P,t}} - v^{-}_{\mathrm{foot,t}}}{v^{-}_{\mathrm{P,t}}}. \tag{S5}$$

These velocities are in a reference frame that is parallel to the tangential and normal directions of the terrain at the point of contact ($\hat{t} - \hat{n}$ frame in figure S2). Point P refers to the contact point in the rigid runner that is coincident with the foot at the time of contact, but whose speed is solely a function of the body's linear and angular momentum.

When the foot speed exactly matches ground speed as seen from the body-fixed frame, $\epsilon_{\mathrm{t}} = 1$ and the tangential collision impulse would be zero. To an external observer, the foot's velocity vector would be exactly normal to the terrain at the point of contact and $v^{-}_{\mathrm{foot,t}} = 0$. If the foot's speed equals that of point P of the rigid runner, then $\epsilon_{\mathrm{t}} = 0$ and the runner experiences a significant tangential collision impulse.

The open-loop and anticipatory strategies differ in how they specify the foot speed. The intended coefficient of restitution ϵ_{tc} for the open-loop strategy is specified under the assumption that the tangential speed of the contact point P equals the initial forward speed, $v^{-}_{\mathrm{P,t}} = v_{x0}$. Furthermore, the open-loop strategy is only aware of the foot speed in a body-fixed frame, and its components relative to gravity, i.e. the components of $\boldsymbol{v}^{-}_{\mathrm{P}} - \boldsymbol{v}^{-}_{\mathrm{foot}}$ in the x-y frame. These capture the three assumptions outlined in the main text (section 2.2). The intended coefficient ϵ_{tc} for the anticipatory strategy makes no such assumption and simply follows equation S5. The respective equations for the anticipatory and open-loop ϵ_{tc} are,

$$\epsilon_{\mathrm{tc}} = \begin{cases} \frac{v^{-}_{\mathrm{P,x}} - v^{-}_{\mathrm{foot,x}}}{v_{x0}} & : \text{open-loop}, \\ \frac{v^{-}_{\mathrm{P,t}} - v^{-}_{\mathrm{foot,t}}}{v^{-}_{\mathrm{P,t}}} & : \text{anticipatory}. \end{cases} \tag{S6}$$

By combining equation (S5) and equation (S6) we find

$$\epsilon_{\mathrm{t}} = \begin{cases} \epsilon_{\mathrm{tc}} \left(\frac{v^{-}_{\mathrm{P,t}} - v^{-}_{\mathrm{foot,t}}}{v^{-}_{\mathrm{P,x}} - v^{-}_{\mathrm{foot,x}}} \right) \left(\frac{v_{x0}}{v^{-}_{\mathrm{P,t}}} \right) & : \text{open-loop}, \\ \epsilon_{\mathrm{tc}} & : \text{anticipatory}. \end{cases} \tag{S7}$$

For small terrain angles θ, the tangential and horizontal components of the foot speed relative to that of point P are nearly equal, i.e. $(\boldsymbol{v}^{-}_{\mathrm{P}} - \boldsymbol{v}^{-}_{\mathrm{foot}}) \cdot \hat{\boldsymbol{t}} \approx (\boldsymbol{v}^{-}_{\mathrm{P}} - \boldsymbol{v}^{-}_{\mathrm{foot}}) \cdot \hat{\boldsymbol{x}}$. Therefore, the main reason for ϵ_{tc} to differ from ϵ_{t} for the open-loop runner is because of the ratio of speed of the contact point $\|\boldsymbol{v}^{-}_{\mathrm{P}}\|$ versus the assumed forward speed v_{x0}. The open-loop relationship thus becomes $\epsilon_{\mathrm{t}} = \epsilon_{\mathrm{tc}} v_{x0} / v^{-}_{\mathrm{P,t}}$, yielding equation (2) used in the main text.

S3.3 Open-loop push-off strategies

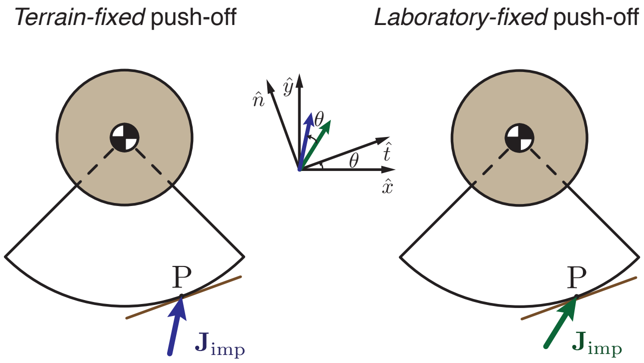

Figure S2: The *terrain-fixed* push-off impulse is the *laboratory-fixed* push-off impulse rotated by the terrain slope angle θ about the contact point.

After the passive collision, the push-off impulse $\boldsymbol{J}_{\text{imp}}$ is applied at the contact point P to propel the runner into the flight phase. Recall that $J_\phi = 0$ for the open-loop push policies that we consider (main text equation (1c)). The push-off impulse $\boldsymbol{J}_{\text{imp}}$ leads to discrete changes in the linear velocity of the center of mass $\boldsymbol{v}_{\text{imp}}$, and angular velocity of the runner ω_{imp}. As discussed in main text section 2.3, a *terrain-fixed* push-off policy or a *laboratory-fixed* push-off policy both satisfy the periodicity criteria on flat ground, namely $\boldsymbol{v}^+_{\text{G,steady}} = (v_{x0}, v_{y0})^{\text{T}}$ and $\omega^+ = 0$, but differ in their behavior on rough terrain. The linear velocity $\boldsymbol{v}^+_{\text{G}}$ and angular velocity ω^+ at take-off under these push-off policies are,

$$\boldsymbol{v}^+_{\text{G}} = \boldsymbol{v}^c_{\text{G}} + \boldsymbol{v}_{\text{imp}}, \quad \omega^+ = \omega^c + \omega_{\text{imp}}, \tag{S8a}$$

$$\text{where } \boldsymbol{v}_{\text{imp}} = \begin{cases} \boldsymbol{R}\begin{pmatrix} \frac{I_{/\text{G}}(1-\epsilon_{\text{t}})v_{\text{x0}}}{I_{/\text{G}}+1} \\ (1-\epsilon_{\text{n}})v_{\text{y0}} \end{pmatrix} & : \textit{terrain-fixed}, \\ \begin{pmatrix} \frac{I_{/\text{G}}(1-\epsilon_{\text{t}})v_{\text{x0}}}{I_{/\text{G}}+1} \\ (1-\epsilon_{\text{n}})v_{\text{y0}} \end{pmatrix} & : \textit{laboratory-fixed}, \end{cases} \tag{S8b}$$

$$\omega_{\text{imp}} = \begin{cases} \frac{(1-\epsilon_{\text{t}})v_{\text{x0}}}{I_{/\text{G}}+1} & : \textit{terrain-fixed}, \\ \frac{(1-\epsilon_{\text{t}})v_{\text{x0}}\cos\theta}{I_{/\text{G}}+1} + \frac{(1-\epsilon_{\text{n}})v_{\text{y0}}\sin\theta}{I_{/\text{G}}} & : \textit{laboratory-fixed}, \end{cases}, \tag{S8c}$$

$$\text{and } \boldsymbol{R} = \begin{pmatrix} \cos\theta & -\sin\theta \\ \sin\theta & \cos\theta \end{pmatrix}. \tag{S8d}$$

S4 Open-loop runners on rough terrain

S4.1 The open-loop passive collision

The tangential collision parameter ϵ_{t} for open-loop runners varies from step-to-step on rough terrains (main text equation (2)) and is thus characterized by distributions (figure S3a) that evolve according to running dynamics described in main text equation (1). These distributions achieve stationarity as seen in the representative case of the ϵ_{t} distribution with $\epsilon_{\text{tc}} = 0.4$, that appears to converge by 5 steps (figure S3a). Mean ϵ_{t} converges by 3 steps to values that are just a little larger than the corresponding ϵ_{tc} values (figure S3c), and show a linear dependence on ϵ_{tc} (figure S3b). The standard deviation of the converged ϵ_{t} distribution increases with ϵ_{tc} (figure S3d).

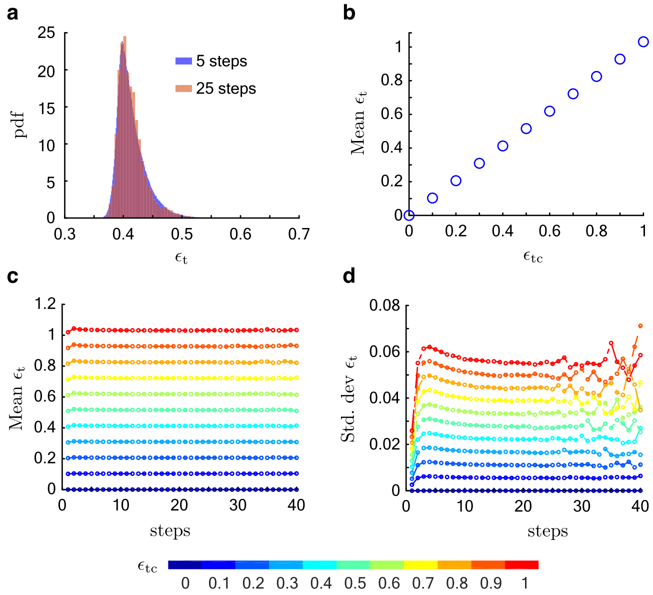

Figure S3: Tangential collisions of open-loop runners with human-like parameters on rough terrain. Monte Carlo simulations performed with 10^6 runners. (**a**) The probability density function of ϵ_t after 5 steps and 25 steps for $\epsilon_{tc} = 0.4$. (**b**) Steady-state mean ϵ_t increases linearly with ϵ_{tc}. (**c**) Mean ϵ_t as a function of step number for different values of ϵ_{tc} (shown in the color bar below) converges by approximately 3 steps. (**d**) Standard deviation of ϵ_t as a function of step number for different values of ϵ_{tc} also reaches a steady-state. However, the rate of convergence is slower at higher values of ϵ_{tc}. The fluctuations at higher step number ($\gtrapprox 25$) are because we are probing the tails of the steps to failure distributions.

The stability benefits of higher mean ϵ_t values (figure S3b) are mitigated by higher fluctuations in ϵ_t at higher ϵ_{tc} values (figure S3d) leading to larger step-to-step fluctuations in body angular momentum. Hence for open-loop runners, mean steps to failure shows a weak dependence on ϵ_{tc} value. In main text section 5, we arrive at this same conclusion via an asymptotic analysis of the series expansion of the orientation change over a single step caused by an unexpected terrain slope perturbation.

S4.2 Additive noise in open-loop strategies

Additive noise in the tangential collision (main text section 3.5) of open-loop runners has little effect on mean steps to failure (figure S4) compared to the same noise intensity applied to anticipatory runners (main text figure 3b). For open-loop runners with human-like parameters, there is no optimal value of ϵ_{tc} (figure S4) unlike the case with anticipatory runners where slight tangential collisions are optimal (main text figure 3b). The relative insensitivity of failure statistics to added noise for the open-loop runner is possibly because ϵ_t varies significantly on rough terrains even in the absence of added noise (figure S3).

S5 Anticipatory runners on rough terrain

In main text section 3.4 we describe how eliminating the tangential collision impulse stabilizes body orientation, but deviations from this strategy quickly lead to a loss in stability. The dominant failure

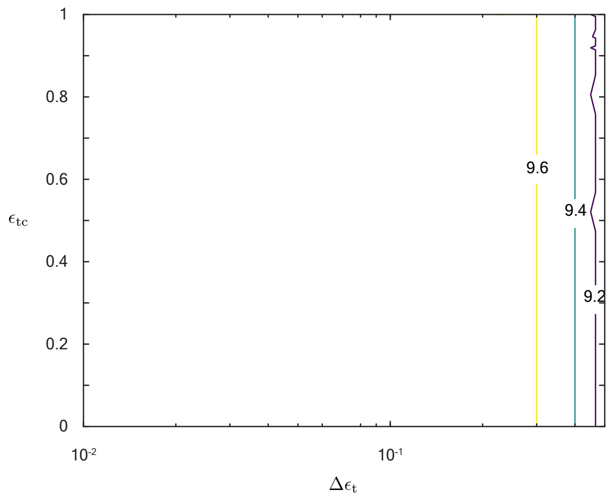

Figure S4: Effect of additive noise in $\epsilon_{\rm tc}$ for open-loop runners with human-like parameters. Contour plot of mean steps to failure as a function of $\epsilon_{\rm tc}$ and $\Delta\epsilon_{\rm t}$. The mean steps to failure is constant as a function of $\epsilon_{\rm tc}$ and $\Delta\epsilon_{\rm t}$ below $\Delta\epsilon_{\rm t} = 0.2$, and shows a small decrease with increasing $\Delta\epsilon_{\rm t}$ when $\Delta\epsilon_{\rm t} > 0.2$. Contours remain nearly parallel to the $\epsilon_{\rm tc}$ axis.

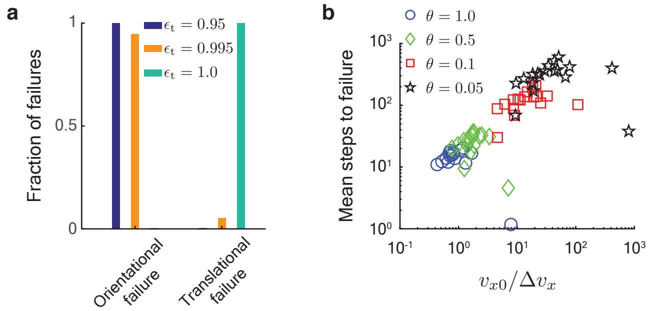

Figure S5: Anticipatory runners on rough terrain. (**a**) Relative frequency of failure modes for anticipatory runners with $\epsilon_{\rm n} = 0$ on rough terrain. The orientation failure bound $\phi_{\rm tol} = \pi/6$ in these simulations and other parameters are the same as those for the human-like runner (main text table 1) (**b**) Mean steps to failure as function of $v_{\rm x0}/\Delta v_x$ for the same range of parameters shown in figure S7, except that $\epsilon_{\rm tc} = 1$ here.

mode changes quickly; anticipatory runners with $\epsilon_{\rm tc} = 1$ fail exclusively due to translational instabilities while anticipatory runners with human-like parameters and $\epsilon_{\rm tc} = 0.95$ fail exclusively due to orientation instabilities (figure S5a). Even runners with $\epsilon_{\rm tc} = 0.995$ predominantly undergoing orientation failures (figure S5a).

The mean steps to failure for an anticipatory runner with $\epsilon_{\rm tc} = 1$ is captured by a single parameter $v_{x0}/\Delta v_x$ (figure S5b). The logic is similar to that in main text section 5 where the scaling analysis is for orientation failures, while here the failure mode is due to losing translational stability (main text figure 1c). For a runner landing on sloped ground with forward speed $v^-_{\rm G,x} = v_{x0}$, the loss in forward speed over a single step due to the terrain perturbation is $\Delta v_x = v_{x0} - v^+_{\rm G,x}$.

Following the logic outlined in main text section 5, the mean steps to failure N should scale as,

$$N \sim v_{x0}/\Delta v_x, \tag{S9a}$$

$$\text{where } \Delta v_x = \sin\theta(v_{y0}(\cos\theta - 1 + \epsilon_\mathrm{n}(\cos\theta + 1)) + v_{x0}\sin\theta(1+\epsilon_\mathrm{n})). \tag{S9b}$$

$$\text{Power series: } \Delta v_x = 2\epsilon_\mathrm{n} v_{y0}\theta + v_{x0}(1+\epsilon_\mathrm{n})\theta^2 + O(\theta^3). \tag{S9c}$$

Equation (S9c) is the power series expansion for Δv_x about $\theta = 0$ to second order in θ. The power series analysis suggests that shallower flight trajectories (reducing v_{y0}) and increasing energy dissipation (reducing ϵ_n) increases the mean steps to translational failure. The implications of shallower flight trajectories have been discussed in the discussion section of the main text. Results from the Monte Carlo simulations in main text figure 3a find that reducing ϵ_n increases mean steps taken for anticipatory runners with $\epsilon_\mathrm{tc} = 1$, consistent with equations (S9b)-(S9c).

S5.1 Forward collision parameter $\hat{\epsilon}_\mathrm{t}$ as a function of ϵ_tc

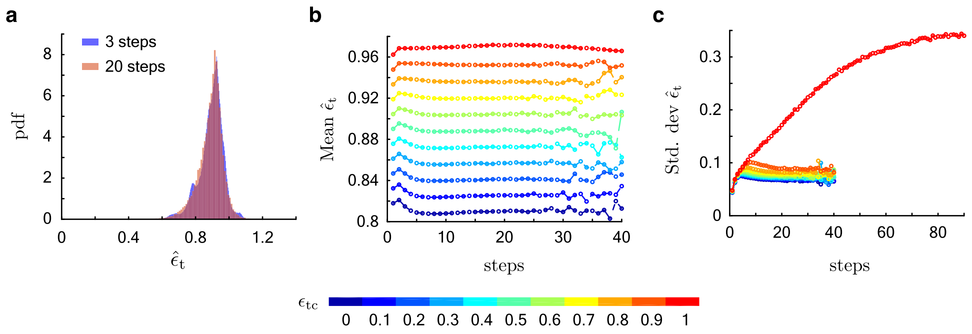

Figure S6: The forward collision parameter $\hat{\epsilon}_\mathrm{t}$ distribution as a function of step number and ϵ_tc. (**a**) The probability density function of $\hat{\epsilon}_\mathrm{t}$ after 3 steps and 20 steps for $\epsilon_\mathrm{tc} = 0.5$. (**b**) Mean $\hat{\epsilon}_\mathrm{t}$ as a function of step number for different values of ϵ_tc (shown in the color bar below). The mean converges by approximately 5 steps for all values of ϵ_tc. (**c**) Standard deviation of $\hat{\epsilon}_\mathrm{t}$ as a function of step number for different values of ϵ_tc. While the standard deviation converges by 15 steps for values of $\epsilon_\mathrm{tc} < 1$, when $\epsilon_\mathrm{tc} = 1$ it increases monotonically, plateauing after approximately 80 steps.

The forward collision parameter $\hat{\epsilon}_\mathrm{t}$ is characterized by a distribution as described in main text section 3.6. The distributions of $\hat{\epsilon}_\mathrm{t}$ with $\epsilon_\mathrm{tc} = 0.5$ after 3 steps and 20 steps are relatively unchanged (figure S6a) in comparison to the distribution of $\hat{\epsilon}_\mathrm{t}$ with $\epsilon_\mathrm{tc} = 1$ (main text figure 4a) where the distribution broadens significantly more as a function of step number. The mean of the distribution for all values of ϵ_tc converges by 5 steps (figure S6b), while the standard deviation for ϵ_tc sufficiently below 1 converges by approximately 15 steps (figure S6c). When $\epsilon_\mathrm{tc} = 1$, or if ϵ_tc is sufficiently close to 1, the standard deviation of the distribution approaches steady state only after 80 steps.

S6 Linear stability analysis: Jordan decomposition of $\boldsymbol{T}_\mathrm{ol}$ and $\boldsymbol{T}_\mathrm{an}$

The linear stability analysis for the open-loop runner proceeds analogously to the analysis for the anticipatory runner discussed in main text section 4. We define a Poincaré section transverse to the runner's trajectory in phase space at the apex of the aerial phase ($v_y = 0$), and a return map $\boldsymbol{f}_\mathrm{ol}$ that maps the state of the runner $\boldsymbol{\psi}_n$ at the apex of the aerial phase at step n, to the state at

the apex of the aerial phase on the following step $\boldsymbol{\psi}_{n+1}$ (main text figure 5). The state $\boldsymbol{\psi}$, return map $\boldsymbol{f}_{\text{ol}}$, and its linearization $\boldsymbol{T}_{\text{ol}}$ are defined by,

$$\boldsymbol{\psi} = \begin{pmatrix} x & y & \phi & v_x & \omega \end{pmatrix}^{\text{T}} \tag{S10a}$$

$$\boldsymbol{\psi}_{n+1} = \boldsymbol{f}_{\text{ol}}(\boldsymbol{\psi}_n), \tag{S10b}$$

$$\Delta\boldsymbol{\psi}_{n+1} = \boldsymbol{T}_{\text{ol}}\Delta\boldsymbol{\psi}_n, \text{ where } \boldsymbol{T}_{\text{ol}} = \frac{\partial \boldsymbol{f}_{\text{ol}}}{\partial \boldsymbol{\psi}}\bigg|_{\boldsymbol{\psi}^*}, \tag{S10c}$$

$$\text{and } \Delta\boldsymbol{\psi} = \boldsymbol{\psi} - \boldsymbol{\psi}^*, \text{ where } \boldsymbol{\psi}^* = \boldsymbol{f}_{\text{ol}}(\boldsymbol{\psi}^*). \tag{S10d}$$

The linearized return maps, $\boldsymbol{T}_{\text{ol}}$ and $\boldsymbol{T}_{\text{an}}$ are non-diagonalizable as discussed in main text section 4. Thus an eigen-factorization of these matrices is not possible, so we perform a Jordan decomposition of $\boldsymbol{T}_{\text{ol}}$ and $\boldsymbol{T}_{\text{an}}$. The Jordan decomposition of $\boldsymbol{T}_{\text{ol}}$ is,

$$\boldsymbol{T}_{\text{ol}} = \boldsymbol{V}_{\text{ol}}\boldsymbol{J}_{\text{ol}}\boldsymbol{V}_{\text{ol}}^{-1}, \tag{S11a}$$

$$\text{where } \boldsymbol{J}_{\text{ol}} = \begin{pmatrix} 1 & 0 & \sqrt{2}\epsilon_{\text{n}} v_{y0} & 0 & 0 \\ 0 & 1 & \frac{\sqrt{2}}{1+I_{/\text{G}}} v_{y0}(I_{/\text{G}}(\epsilon_{\text{n}}-1)-\epsilon_{\text{n}}) & 0 & 0 \\ 0 & 0 & 1 & 0 & 0 \\ 0 & 0 & 0 & \epsilon_{\text{n}}(2\epsilon_{\text{n}}-1) & 0 \\ 0 & 0 & 0 & 0 & 0 \end{pmatrix}, \tag{S11b}$$

$$\text{and } \boldsymbol{V}_{\text{ol}} = \begin{pmatrix} 0 & 1 & 0 & 0 & \frac{(1-I_{/\text{G}}-4\epsilon_{\text{n}})v_{\text{y}0}I_{/\text{G}}}{1+I_{/\text{G}}} \\ 0 & 0 & 0 & 1 & 0 \\ 1 & 0 & 0 & 0 & -v_{\text{y}0} \\ 0 & 0 & \frac{-1}{\sqrt{2}} & 0 & I_{/\text{G}} \\ 0 & 0 & \frac{1}{\sqrt{2}} & 0 & 1 \end{pmatrix}. \tag{S11c}$$

The linearized return map $\boldsymbol{T}_{\text{ol}}$ has rank 4, with two stable eigenvalues; $\lambda = 0$ and $\lambda = \epsilon_{\text{n}}(2\epsilon_{\text{n}} - 1)$. The second stable eigenvalue corresponds to perturbations to the height of the apex of the aerial phase. The remaining eigenvalues are $\lambda = 1$ which are associated with eigenvectors $\boldsymbol{\nu}_1$, $\boldsymbol{\nu}_2$, and generalized eigenvector $\boldsymbol{\nu}_3$, the same as the eigenvectors and generalized eigenvector associated with eigenvalues $\lambda = 1$ for $\boldsymbol{T}_{\text{an}}$ (main text equation (7a)). Thus, a perturbation in the subspace spanned by these vectors $\Delta\boldsymbol{\psi}_0$ displays the same scaling with step number n as described in main text equation (8) for the anticipatory runner. These relationships for the open-loop runner are summarized as follows,

$$\Delta\boldsymbol{\psi}_0 = \sum_{k=1}^{3} \alpha_k \nu_k, \;\; \Delta\boldsymbol{\psi}_n = n\alpha_3(a_1\boldsymbol{\nu}_1 + a_2\boldsymbol{\nu}_2) + \Delta\boldsymbol{\psi}_0, \tag{S12a}$$

$$\Delta\boldsymbol{\psi}_n \approx n\;\alpha_3 \begin{pmatrix} \frac{\sqrt{2}}{1+I_{/\text{G}}} v_{y0}(I_{/\text{G}}(\epsilon_{\text{n}}-1)-\epsilon_{\text{n}}) \\ 0 \\ \sqrt{2}\epsilon_{\text{n}} v_{y0} \\ 0 \\ 0 \end{pmatrix}, \text{ for } n \gg 1, \tag{S12b}$$

$$\text{and substituting } a_1 = \sqrt{2}\epsilon_{\text{n}} v_{y0}, \text{ and } a_2 = \frac{\sqrt{2}}{1+I_{/\text{G}}} v_{y0}(I_{/\text{G}}(\epsilon_{\text{n}}-1)-\epsilon_{\text{n}}), \tag{S12c}$$

which are the off-diagonal terms of $\boldsymbol{J}_{\text{ol}}$ (equation S11b). While the growth of the instability shows the same dependence on step number n as the anticipatory runner (main text equation (8)), the

value of a_2 differs, i.e. the projection onto $\boldsymbol{\nu}_2$ of a perturbation along $\boldsymbol{\nu}_3$ upon action of the return map $\boldsymbol{T}_{\mathrm{ol}}$.

The stability of the anticipatory runner is discussed in detail in main text section 4. The Jordan decomposition of $\boldsymbol{T}_{\mathrm{an}}$ when $\epsilon_{\mathrm{tc}} < 1$ is,

$$\boldsymbol{T}_{\mathrm{an}} = \boldsymbol{V}_{\mathrm{an}} \boldsymbol{J}_{\mathrm{an}} \boldsymbol{V}_{\mathrm{an}}^{-1}, \tag{S13a}$$

$$\text{where } \boldsymbol{J}_{\mathrm{an}} = \begin{pmatrix} 1 & 0 & \sqrt{2}\epsilon_{\mathrm{n}} v_{\mathrm{y0}} & 0 & 0 \\ 0 & 1 & -\sqrt{2}\epsilon_{\mathrm{n}} v_{\mathrm{y0}} & 0 & 0 \\ 0 & 0 & 1 & 0 & 0 \\ 0 & 0 & 0 & \epsilon_{\mathrm{n}}(2\epsilon_{\mathrm{n}} - 1) & 0 \\ 0 & 0 & 0 & 0 & \epsilon_{\mathrm{t}} \end{pmatrix}, \tag{S13b}$$

$$\text{and } \boldsymbol{V}_{\mathrm{an}} = \begin{pmatrix} 0 & 1 & 0 & 0 & \frac{(1+\epsilon_{\mathrm{t}}(2\epsilon_{\mathrm{n}}-1)) I_{/\mathrm{G}} v_{\mathrm{y0}}}{\epsilon_{\mathrm{t}}-1} \\ 0 & 0 & 0 & 1 & 0 \\ 1 & 0 & 0 & 0 & \frac{(1+\epsilon_{\mathrm{t}}(2\epsilon_{\mathrm{n}}-1)) v_{\mathrm{y0}}}{\epsilon_{\mathrm{t}}-1} \\ 0 & 0 & \frac{-1}{\sqrt{2}} & 0 & I_{/\mathrm{G}} \\ 0 & 0 & \frac{1}{\sqrt{2}} & 0 & 1 \end{pmatrix}. \tag{S13c}$$

The anticipatory runner has a full rank return map $\boldsymbol{T}_{\mathrm{an}}$, and stable eigenvalues $\lambda = \epsilon_{\mathrm{n}}(2\epsilon_{\mathrm{n}} - 1)$, which is identical to the open-loop runner, and $\lambda = \epsilon_{\mathrm{t}}$ which differs from the open-loop runner. The off-diagonal elements of $\boldsymbol{J}_{\mathrm{an}}$ are $a_1 = \sqrt{2}\epsilon_{\mathrm{n}} v_{\mathrm{y0}}$ and $a_2 = -\sqrt{2}\epsilon_{\mathrm{n}} v_{\mathrm{y0}}$ (equation S13b), which correspond to the projection onto $\boldsymbol{\nu}_1$ and $\boldsymbol{\nu}_2$ respectively, of a perturbation along $\boldsymbol{\nu}_3$ upon action of the return map $\boldsymbol{T}_{\mathrm{an}}$.

When $\epsilon_{\mathrm{tc}} = \epsilon_{\mathrm{t}} = 1$ for the anticipatory runner, the linearization of $\boldsymbol{f}_{\mathrm{an}}$ about $\boldsymbol{\psi}^*$ (main text equations 6) yields a different form for $\boldsymbol{T}_{\mathrm{an}}$. The Jordan decomposition of $\boldsymbol{T}_{\mathrm{an}}$ when $\epsilon_{\mathrm{tc}} = 1$ is,

$$\boldsymbol{T}_{\mathrm{an}} = \boldsymbol{V}_{\mathrm{an}} \boldsymbol{J}_{\mathrm{an}} \boldsymbol{V}_{\mathrm{an}}^{-1}, \tag{S14a}$$

$$\text{where } \boldsymbol{J}_{\mathrm{an}} = \begin{pmatrix} 1 & 2\epsilon_{\mathrm{n}} v_{y0} & 0 & 0 & 0 \\ 0 & 1 & 0 & 0 & 0 \\ 0 & 0 & 1 & 2\epsilon_{\mathrm{n}} v_{y0} & 0 \\ 0 & 0 & 0 & 1 & 0 \\ 0 & 0 & 0 & 0 & \epsilon_{\mathrm{n}}(2\epsilon_{\mathrm{n}} - 1) \end{pmatrix}, \tag{S14b}$$

$$\text{and } \boldsymbol{V}_{\mathrm{an}} = \begin{pmatrix} 0 & 0 & 1 & 0 & 0 \\ 0 & 0 & 0 & 0 & 1 \\ 1 & 0 & 0 & 0 & 0 \\ 0 & 0 & 0 & 1 & 0 \\ 0 & 1 & 0 & 0 & 0 \end{pmatrix}. \tag{S14c}$$

There is a single stable eigenvalue of $\lambda = \epsilon_{\mathrm{n}}(2\epsilon_{\mathrm{n}} - 1)$ which is identical to that of the open-loop runner and the anticipatory runner with $\epsilon_{\mathrm{tc}} < 1$. The remaining 4 eigenvalues are $\lambda = 1$ which are associated with eigenvectors $\boldsymbol{\nu}_1$, $\boldsymbol{\nu}_2$ and generalized eigenvectors $\boldsymbol{\nu}_3$ and $\boldsymbol{\nu}_4$ (main text equations (9a), columns of $\boldsymbol{V}_{\mathrm{an}}$ from equation (S14c)). Perturbations along $\boldsymbol{\nu}_3$ project onto $\boldsymbol{\nu}_1$, and perturbations along $\boldsymbol{\nu}_4$ project onto $\boldsymbol{\nu}_2$, upon action of the return map $\boldsymbol{T}_{\mathrm{an}}$ (equation (S14b)). The off-diagonal elements of $\boldsymbol{J}_{\mathrm{an}}$ are $a_1 = a_2 = 2\epsilon_{\mathrm{n}} v_{y0}$.

S7 Scaling analysis of orientation failures

In main text section 5 we show that the orientation failure bound ϕ_{tol} and the orientation change over a single step from encountering an unexpected terrain slope $\phi_\bullet$ predict the mean steps to failure

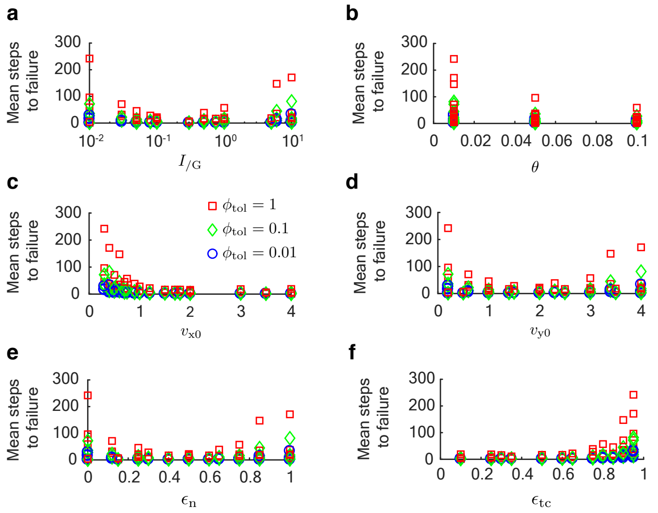

Figure S7: Mean steps to failure for the anticipatory runner ($\epsilon_{\mathrm{tc}} < 1$) plotted against all other parameters: $I_{/G}, \theta, v_{\mathrm{y0}}, \epsilon_{\mathrm{n}}, v_{\mathrm{x0}}, \epsilon_{\mathrm{tc}}$. Here, θ refers to the typical slope angle the runner would encounter, defined as $\theta = h/\lambda$, where λ is the grid spacing of the terrain and $[-h, h]$ is the range of heights of the terrain (section S1.3).

N, as $N \sim \phi_{\mathrm{tol}}/\phi_{\bullet}$. The many parameters that define the runner and its gait do not separately predict mean steps to failure as accurately as the parameter $\phi_{\mathrm{tol}}/\phi_{\bullet}$ (figure S7).

The change in orientation over one step can be calculated using the vertical velocity $v_{y,\bullet}^{+}$ and angular velocity $\omega_{\bullet}^{+}$ at take-off as $\phi_{\bullet} = 2v_{y,\bullet}^{+}\omega_{\bullet}^{+}$ ($\bullet$ = 'ol' for open-loop and 'an' for anticipatory). For a runner landing on a terrain patch angled at θ with respect to the horizontal, with center of mass velocity $\boldsymbol{v}_{\mathrm{G}}^{-} = \begin{pmatrix} v_{x0} & -v_{y0} \end{pmatrix}^{\mathrm{T}}$ and angular velocity $\omega^{-} = 0$, the take-off velocities $v_{y,\bullet}^{+}, \omega_{\bullet}^{+}$, and orientation change $\phi_{\bullet}$, are given by

$$\omega_{\mathrm{an}}^{+} = \frac{(1-\epsilon_{\mathrm{tc}})(v_{x0}(1-\cos\theta) + v_{y0}\sin\theta)}{1+I_{/\mathrm{G}}}, \tag{S15a}$$

$$\omega_{\mathrm{ol}}^{+} = \frac{v_{x0}(1-\cos\theta) + v_{y0}\sin\theta}{1+I_{/\mathrm{G}}}, \tag{S15b}$$

$$v_{y,\mathrm{an}}^{+} = v_{y0} + \frac{(1+\epsilon_{\mathrm{n}} + I_{/\mathrm{G}}\epsilon_{\mathrm{n}})(v_{x0}\sin 2\theta - v_{y0}(1-\cos 2\theta))}{2(1+I_{/\mathrm{G}})}, \tag{S15c}$$

$$v_{y,\mathrm{ol}}^{+} = v_{y,\mathrm{an}} + \frac{2\epsilon_{\mathrm{tc}} v_{\mathrm{x0}}\sin\theta}{2(1+I_{/\mathrm{G}})}, \tag{S15d}$$

$$\begin{aligned} \phi_{\mathrm{an}} &= \frac{1-\epsilon_{\mathrm{tc}}}{(1+I_{/\mathrm{G}})^2} A\big(B + (\epsilon_{\mathrm{tc}}-1)C + (D + I_{/\mathrm{G}}\epsilon_{\mathrm{tc}})E + F - I_{/\mathrm{G}}\epsilon_{\mathrm{tc}} v_{y0}\big), \\ \phi_{\mathrm{ol}} &= \frac{1}{(1+I_{/\mathrm{G}})^2} A\big(B + (2\epsilon_{\mathrm{tc}}-1)C + DE + F\big), \\ \text{where } A &= v_{y0}\sin\theta + v_{x0}(1-\cos\theta),\ B = 2(1+I_{/\mathrm{G}})(1-\epsilon_{\mathrm{n}})v_{y0}\cos\theta, \\ C &= 2I_{/\mathrm{G}} v_{x0}\sin\theta,\ D = 1 + \epsilon_{\mathrm{n}} + I_{/\mathrm{G}}\epsilon_{\mathrm{n}}, \\ E &= v_{y0}\cos 2\theta + v_{x0}\sin 2\theta,\ F = (I_{/\mathrm{G}}\epsilon_{\mathrm{n}} + \epsilon_{\mathrm{n}} - 1)v_{y0}. \end{aligned} \tag{S15e}$$

The parametric dependence of $\phi_{\mathrm{tol}}/\phi_{\bullet}$ ($\phi_{\bullet}$ defined in equation (S15e)) on ϵ_{tc} and ϵ_{n} is captured by a power series approximation of $\phi_{\bullet}$ to second order in θ (main text equations (11a)-(11b)). The

contour plots of $\phi_{\rm tol}/\phi_\bullet$ shown here using the truncated power series form of $\phi_\bullet$ (figure S8) display the same qualitative trends as the ones in main text figure 7 which use the complete expression for $\phi_\bullet$ (equation (S15e)). Therefore, we analyze failure statistics and runner morphology in main text section 5 and section 6 using the truncated power series approximation for $\phi_\bullet$.

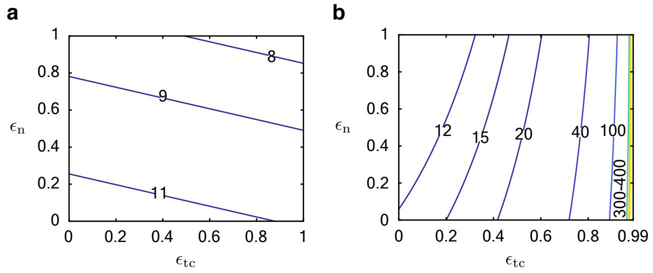

Figure S8: Contour plots of (**a**) $\phi_{\rm tol}/\phi_{\rm ol}$ and (**b**) $\phi_{\rm tol}/\phi_{\rm an}$ as a function of $\epsilon_{\rm n}$ and $\epsilon_{\rm tc}$, where the form of $\phi_\bullet$ shown in the power series expansion to second order in θ has been used (main text equations (11a)-(11b)). The values of the contours differ between the plots shown here and in the main text figure 7 but the qualitative trends are the same. The same parameter values are used in both these calculation, $v_{y0} = 0.2, I_{/\rm G} = 0.15, v_{x0} = 1, \theta = 0.3, \phi_{\rm tol} = 1$. For the anticipatory runner, maximum $\epsilon_{\rm tc} = 0.99$ to ensure that the calculation is limited to the region where runners primarily undergo orientation failures (figure S5a).

S8 Steps to failure statistics

All the numerically estimated distributions of the steps to failure show similar qualitative features. They are unimodal with a long tail, and are relatively insensitive to changes in the terrain height distribution (figure S1, main text figure 2e). The similarity of the distributions along with the results of the linear stability analysis (main text section 4) suggests that the stochastic dynamics underlying these distributions may be characterized by a small number of parameters.

The linear stability analysis shows that perturbations from the ground do not directly affect linear or angular velocities, but affect position variables (main text section 4). For orientation variables, this implies that the rate of change of the angular velocity is zero. However, the terrain introduces perturbations at every step. When the correlation length of the random terrain is much smaller than a step length, like in the Monte Carlo simulations, the step-to-step perturbations may be treated as uncorrelated random perturbations. Irrespective of the distribution from which the random terrain is constructed, we make a heuristic appeal to the central limit theorem and posit that the net effect of the terrain perturbations is simply a Gaussian random forcing. Thus the stochastic step-to-step model for the orientation is that the discrete second derivative of the angle ϕ is forced by a Gaussian random variable:

$$\phi(n+2) - 2\phi(n+1) + \phi(n) = w, \tag{S16a}$$

$$\text{where } w \sim \mathcal{N}(0, \sigma^2), \tag{S16b}$$

The random forcing w is drawn from a Gaussian with mean $< w >= 0$ and variance $< w^2 >= \sigma^2$. In the absence of noise, the solution to the second-order difference equation yields $\phi(n) = n(\phi(1) - \phi(0)) + \phi(0)$. Thus the orientation of the runner ϕ grows linearly with step number n in response to an initial perturbation $(\phi(1) - \phi(0))$ as found in main text section 4.

With initial conditions $\phi(1) = \phi(0) = 0$, the probability distribution of ϕ after n steps is given by

$$p(\phi, n) = \frac{e^{-\frac{\phi^2}{2\sigma^2 f(n)}}}{\sqrt{2\pi\sigma^2 f(n)}}, \tag{S17a}$$

$$\text{where } f(n) = \frac{n(n+1)(2n+1)}{6}. \tag{S17b}$$

$$\text{When } n \gg 1,\ f(n) \sim \frac{n^3}{3}. \tag{S17c}$$

The probability distribution $p(\phi, n)$ is Gaussian with $<\phi>= 0, <(\phi- <\phi>)^2>\sim n^3$ if $n >> 1$.

Orientation failures occur when a runner's orientation exceeds ϕ_{tol}. Thus, the probability of having failed by n steps, which we denote with $p_{\text{fall}}(n)$, is given by

$$p_{\text{fall}}(n) = p(|\phi| > \phi_{\text{tol}}, n), \tag{S18a}$$

$$\implies p_{\text{fall}}(n) = 1 - \text{erf}\left(\frac{\phi_{\text{tol}}}{\sqrt{2\sigma^2 f(n)}}\right), \tag{S18b}$$

$$\text{where erf}(x) = \frac{1}{\sqrt{\pi}}\int_{-x}^{x} e^{-u^2}du, \tag{S18c}$$

is the error function. The probability of failing at step number n, denoted by $p_{\text{steps}}(n)$, is given by the probability of taking $n-1$ steps without failing, and then failing at step number n, i.e. $p_{\text{steps}}(n) = p(|\phi| > \phi_{\text{tol}}, n \Big| |\phi| < \phi_{\text{tol}}, n-1)$. This is equivalent to the probability flux at ϕ_{tol} at step n, given by

$$p_{\text{steps}}(n) = p_{\text{fall}}(n) - p_{\text{fall}}(n-1), \tag{S19a}$$

$$\implies p_{\text{steps}}(n) = \text{erf}\left(\frac{\phi_{\text{tol}}}{\sqrt{2\sigma^2 f(n-1)}}\right) - \text{erf}\left(\frac{\phi_{\text{tol}}}{\sqrt{2\sigma^2 f(n)}}\right). \tag{S19b}$$

The mean steps to failure N are thus given by

$$N = \sum_{n=0}^{\infty} n p_{\text{steps}}(n), \tag{S20a}$$

$$\implies N = \sqrt{\frac{6}{\pi}} \sum_{i=1}^{\infty} -1^{i+1}\left(\frac{3}{2}\right)^i \zeta\left(\frac{3(2i-1)}{2}\right)\frac{(\phi_{\text{tol}}/\sigma)^{2i-1}}{(i-1)!}, \tag{S20b}$$

We use the approximation $f(n) = n^3/3$ and $\zeta(p)$ is the Riemann-zeta function defined by $\zeta(p) = \sum_{i=0}^{\infty} 1/i^p$ for $p > 1$. The mean steps to failure depend on a single parameter, ϕ_{tol}/σ, the ratio of the tolerance-angle ϕ_{tol} to the intensity of the external noisy forcing w. We numerically solve for the infinite sum in equation (S20b) for figure S9c,d.

The probability of failing at step n, i.e. $p_{\text{steps}}(n)$ (figure S9a), and its cumulative distribution $p_{\text{fall}}(n)$ (figure S9b) are qualitatively similar to the numerically computed steps to failure distributions shown in figure S1 and main text figure 2e, and the cumulative distributions shown in main text figure 2a,b, in that they are unimodal with long tails.

We compare the mean of the numerically computed steps to failure distributions and the analytically derived mean from the stochastic model (figure S9c,d). The standard deviation of the noise

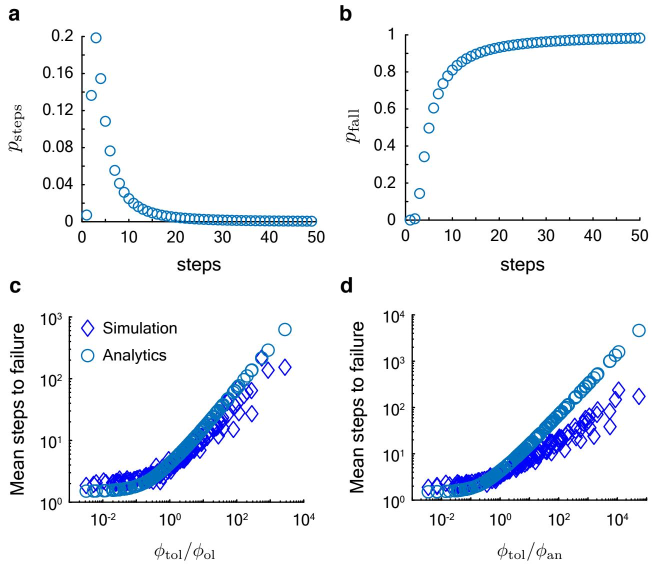

Figure S9: Steps to failure statistics of the single parameter stochastic model. (**a**) $p_{\rm steps}$ from equation (S19) and (**b**) $p_{\rm fall}$ from equation (S18) plotted against steps taken n with human-like parameter values for $\phi_{\rm tol}$ and $\sigma = \phi_{\rm ol}$ (main text table 1). The mean of this distribution 9.6, is similar to the numerically computed mean steps to failure for the open-loop runner with human-like parameters which is 9.61 ± 0.01 (figure S1c). However, for a wider range of $\phi_{\rm tol}/\phi_\bullet$ values, the mean of the analytical model (open circles) for (**c**) Open-loop runners and (**d**) Anticipatory runners deviates from mean steps to failure from the Monte Carlo simulations (open diamonds, main text figure 6). The parameters values used for $\phi_{\rm tol}$ and $\phi_\bullet$ are shown in figure S7.

from the terrain is equated to the orientation change in a single step due to the terrain according to $\sigma = \phi_\bullet$ (main text section 5) since $\phi_\bullet$ sets the scale for orientation change over a single step due to an unexpected terrain slope (main text section 5). The mean steps to failure in both the Monte Carlo simulations and the analytical Markov chain model show power law dependence as a function of $\phi_{\rm tol}/\phi_\bullet$, but with different exponents (figure S9c,d).

S9 Effect of changing runner size relative to terrain grid spacing

The size of the runner r_ℓ relative to the grid spacing of the terrain λ determines the range of terrain slopes accessed by the runner (main text section 6, figure S10). For example, if $\lambda/r_\ell = 1$, the range of terrain slopes encountered by the runner is nearly the same as the slope distribution of the terrain itself (figure S10a). By decreasing this ratio to $\lambda/r_\ell = 0.01$ while keeping the terrain slope distribution constant, we find that the range of slopes encountered by the runner is significantly lower (figure S10b). We quantify this trend using the parameter $\sigma_{\rm runner}/\sigma_{\rm terrain}$ where $\sigma_{\rm runner}$ is the standard deviation of the slope distribution encountered by the runner, and $\sigma_{\rm terrain}$ is the standard deviation of the terrain slope distribution. The parameter $\sigma_{\rm runner}/\sigma_{\rm terrain}$ approaches 1 as λ/r_ℓ increases (figure S10c).

The mean slope encountered by the runner is always greater than zero (figure S10d) even though the slope distribution of the terrain has a zero mean. As the range of slopes encountered by the runner increases, the mean slope encountered by the runner deviates further from zero (figure S10d). This is consistent with the observation from the Monte Carlo simulations that runners slow down on rough terrain since the effect of the mean slope encountered is to redirect forward momentum

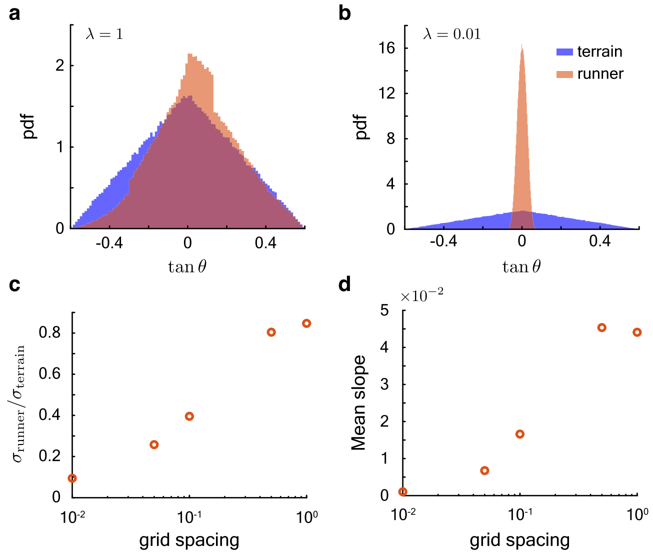

Figure S10: Effect of varying the ratio of leg length r_ℓ to terrain grid spacing λ. The probability density function of the terrain slope $(\tan\theta)$ encountered by the open-loop runner (orange) with human-like parameters is plotted for grid spacing (**a**) $\lambda = r_\ell$ and (**b**) $\lambda = 0.01r_\ell$. The height distribution of the terrain grid points was adjusted so that the terrain's slope distribution (blue) was the same in all simulations. The number of steps used to generate these probability density functions was $\sim 10^6$. (**c**) Shows the standard deviation of the slope encountered by the runner relative to the standard deviation of the terrain's slope distribution. (**d**) The mean slope encountered by the runner as a function of grid spacing λ.

into vertical momentum.

S10 Monte Carlo simulations with a *laboratory-fixed* push-off

The change in angular velocity due to the push-off impulse $\boldsymbol{J}_{\rm imp}$ applied as per the *laboratory-fixed* push-off policy destabilizes the runner as it exacerbates angular momentum fluctuations that arise due to variations in terrain slope angle θ. When $\theta > 0$, the runner collides with lower tangential velocity compared to flat ground, resulting in a smaller clockwise pitching moment than if the runner were colliding with a flat terrain patch. Yet the clockwise pitching moment induced by the the push-off impulse $\boldsymbol{J}_{\rm imp}$ is larger than what it would be on flat ground (equation S8d), causing the runner to spin excessively in the clockwise direction. Conversely, when $\theta < 0$, the runner lands at a shallower angle, increasing the tangential collision impulse and inducing a larger clockwise angular impulse on the runner compared to what it would experience when $\theta = 0$. However, the push-off impulse induces a smaller counter-clockwise change in angular velocity at push-off $\omega_{\rm imp}$ relative to flat ground (equation S8d), causing the runner to spin in the anti-clockwise direction. In contrast, the angular velocity change due to the push-off impulse under the *terrain-fixed* push-off policy is independent of the terrain slope θ (equation S8d). As we have seen in the main text this push-off cannot stabilize the runner. However, it does not amplify angular momentum fluctuations caused by slope variations like the *laboratory-fixed* push-off policy. Thus, the *laboratory-fixed* push-off policy leads to orientation failures in fewer steps compared to the *terrain-fixed* push-off policy.

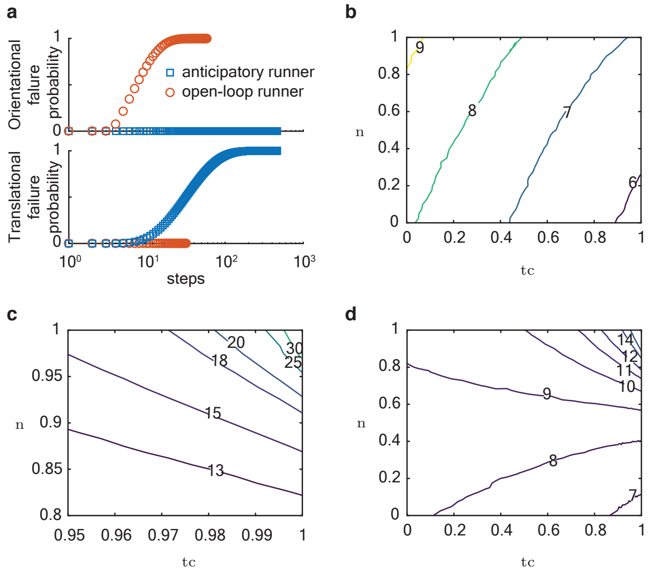

Figure S11: Runners with a *laboratory-fixed* push-off policy: effect of energy dissipation and tangential collisions. (**a**) Open-loop runners with human-like parameters and $\epsilon_{\mathrm{tc}} = 0, \epsilon_{\mathrm{n}} = 1$ undergo orientation failures on rough terrain, while human-like anticipatory runners with $\epsilon_{\mathrm{tc}} = 1, \epsilon_{\mathrm{n}} = 1$ maintain orientation but lose forward momentum. (**b**) Contour map of mean steps to failure for the human-like open-loop runner as a function of ϵ_{n} and ϵ_{tc}. (**c**) Contour map of the mean steps to failure for the anticipatory runner as a function of ϵ_{n} and ϵ_{tc} zoomed into the region near the maximum steps taken where contour spacing is small. (**d**) Away from the maximum, contour spacing increases for the anticipatory runner demonstrating reduced sensitivity to changes in ϵ_{n} and ϵ_{tc}. The $\epsilon_{\mathrm{tc}} = 0$ strategy is identical between the open-loop and anticipatory runners like with the *terrain-fixed* push-off policy.

S10.1 Open-loop runners

Open-loop runners with a *laboratory-fixed* push-off and human-like parameters only fail due to losing orientation stability (figure S11a). Increasing energy dissipation in the direction normal to the terrain surface reduces mean steps taken by these open-loop runners. This trend contrasts that observed with the *terrain-fixed* push-off where increasing normal energy dissipation increases the steps taken (main text figure 2d). The increase in mean steps taken with the *laboratory-fixed* push-off is at most 1 for a 100% reduction in energy dissipation, from $\epsilon_{\mathrm{n}} = 0$ to $\epsilon_{\mathrm{n}} = 1$ at a fixed value of ϵ_{tc} (figure S11b).

Reducing ϵ_{tc} increases steps taken (figure S11b), unlike the trend observed with the *terrain-fixed* push-off policy where changing ϵ_{tc} had negligible effect on the mean steps to failure (main text figure 2d). Open-loop runners increase mean steps taken by 2 when ϵ_{tc} is reduced from 1 to 0 while holding ϵ_{n} fixed at any value (figure S11b).

Open-loop runners with a *laboratory-fixed* push-off policy perform best when $\epsilon_{\mathrm{n}} = 1, \epsilon_{\mathrm{tc}} = 0$ and worst when $\epsilon_{\mathrm{n}} = 0, \epsilon_{\mathrm{tc}} = 1$. The difference in mean steps taken at these extremes is just 3 steps. The maximum mean steps taken by the open-loop runner with a *laboratory-fixed* push-off is 9 steps (figure S11b), the same as the minimum number of steps taken by the open-loop runner with a *terrain-fixed* push-off policy (main text figure 2d). Thus, compared to the *terrain-fixed* push-off policy, open-loop runners with the *laboratory-fixed* push-off policy fare worse, and display differing trends in how stability is determined by the collision parameters, ϵ_{n} and ϵ_{tc}. However,

similar to the *terrain-fixed* push-off policy, varying parameters governing the passive collision has a small effect on the mean steps to failure compared to similar changes in collision parameters in anticipatory runners.

S10.2 Anticipatory runners

Anticipatory runners with a *laboratory-fixed* push-off policy increase mean steps taken as energy dissipation in the direction normal to the terrain surface is reduced. This is in contrast to the trend observed for anticipatory runners with the *terrain-fixed* push-off policy, where increasing normal energy dissipation increases mean steps to failure (main text figure 3a). Anticipatory runners fail by losing orientation stability except when $\epsilon_{\mathrm{tc}} = 1$ and $\epsilon_{\mathrm{n}} = 1$ (figure S11a), in contrast to the *terrain-fixed* push-off policy where anticipatory runners maintain orientation when $\epsilon_{\mathrm{tc}} = 1$, and regardless of the value of ϵ_{n} (main text figure 3a). At $\epsilon_{\mathrm{tc}} = 1$, anticipatory runners with a *laboratory-fixed* push-off, increase mean steps taken by over 4-fold, from 7 to over 30 for a 100% reduction in normal energy dissipation from $\epsilon_{\mathrm{n}} = 0$ to $\epsilon_{\mathrm{n}} = 1$ (figure S11c).

Reducing the tangential collisional impulse at landing ϵ_{tc} increases mean steps to failure for the anticipatory runner with a *laboratory-fixed* push-off if ϵ_{n} is sufficiently high, while showing the opposite trend below that ϵ_{n} value (figure S11d). This is in contrast to the trend observed in anticipatory runners with the *terrain-fixed* push-off policy where mean steps taken always increases when ϵ_{tc} is reduced (main text figure 3a).

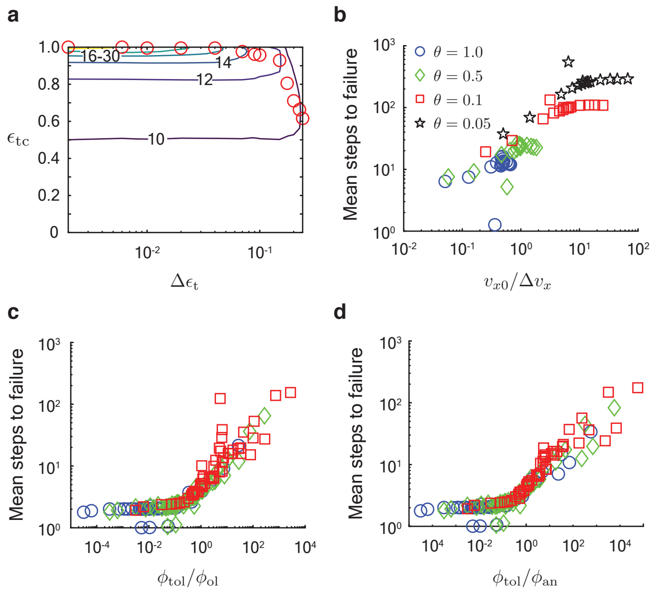

Figure S12: Effect of sensorimotor noise on stability, and scaling analysis of mean steps to failure. (**a**) Contour map of mean steps taken as a function of ϵ_{tc} and $\Delta\epsilon_{\mathrm{t}}$ shows that mean steps to failure reduces with increasing sensorimotor noise $\Delta\epsilon_{\mathrm{t}}$. Scuffing the ground upon landing is optimal as noise intensity $\Delta\epsilon_{\mathrm{t}}$ is increased. Red circles denote the optimal ϵ_{tc} value for a given $\Delta\epsilon_{\mathrm{t}}$. In these simulations $\epsilon_{\mathrm{n}} = 1$. (**b**) For the anticipatory runner with $\epsilon_{\mathrm{tc}} = 1, \epsilon_{\mathrm{n}} = 1$, the mean steps to translational failure are predicted by the parameter $v_{x0}/\Delta v_x$. The values of the remaining parameters used in this simulation are shown in figure S7. Mean steps to orientation failure are captured by the parameter (**c**) $\phi_{\mathrm{tol}}/\phi_{\mathrm{ol}}$ for the open-loop runner, and by (**d**) $\phi_{\mathrm{tol}}/\phi_{\mathrm{an}}$, for the anticipatory runner. The parameter values used for these simulations are also shown in figure S7.

Anticipatory runners with a *laboratory-fixed* push-off policy perform best when $\epsilon_{\mathrm{tc}} = 1, \epsilon_{\mathrm{n}} = 0$ and worst when $\epsilon_{\mathrm{tc}} = 1, \epsilon_{\mathrm{n}} = 0$. The *laboratory-fixed* push-off policy is consistently worse in terms of stability than the *terrain-fixed* push-off policy for a given pair of $\epsilon_{\mathrm{tc}}, \epsilon_{\mathrm{n}}$ values. At $\epsilon_{\mathrm{tc}} = 1, \epsilon_{\mathrm{n}} = 1$ both push-off policies are identical, as this corresponds to $\boldsymbol{J}_{\mathrm{imp}} = 0$ in both.

Running with slight non-zero tangential collisions is optimal in the presence of sensorimotor noise (figure S12a). Sensorimotor noise is modeled as additive noise to ϵ_{tc} as described in main text section 3.5. This trend is similar to that found with the *terrain-fixed* push-off policy (main text figure 3b).

S10.3 Scaling analysis of mean steps taken

The mean steps to failure N for the steps to orientation failure on rough terrains are predicted by the orientation change over a single step relative to the failure bound $\phi_{\mathrm{tol}}/\phi_{\bullet}$, $N_{\bullet} \sim \phi_{\mathrm{tol}}/\phi_{\bullet}$ (figure S12c,d). The loss of forward speed relative to initial forward speed $(v_{x0}/\Delta v_x)$ predicts the mean steps to translational failure $N \sim v_{x0}/\Delta v_x$ for anticipatory runners with $\epsilon_{\mathrm{tc}} = 1$ and $\epsilon_{\mathrm{n}} = 1$ (figure S12b). These trends are similar to those observed for the runner with a *terrain-fixed* push-off policy (main text figure 6), although there is a greater spread in the data with the *laboratory-fixed* push-off.

Supplementary references

[1] **Erdmann, W. S.** (1999). Geometry and inertia of the human body-review of research. *Acta of Bioengineering and biomechanics* **1**, 23–35.

[2] **Alexander, R., Ker, R., Bennet, M., Bibby, S. and Kester, R.** (1987). The spring in the arch of the human foot. *Nature* **325**, 147–149.

[3] **Cavagna, G. and Kaneko, M.** (1977). Mechanical work and efficiency in level walking and running. *The Journal of physiology* **268**, 467.

[4] **Cavagna, G., Saibene, F. and Margaria, R.** (1964). Mechanical work in running. *Journal of applied physiology* **19**, 249–256.

[5] **Dhawale, N. and Venkadesan, M.** (2018). Energetics and stability of running on rough terrains. American Society of Biomechanics, Rochester, MN.

[6] **Dhawale, N., Mandre, S. and Venkadesan, M.** (2015). Running on rough terrains: Energetics and stability. Dynamic Walking, Columbus, Ohio.

[7] **Jenkins, M. and Traub, J. F.** (1970). A three-stage algorithm for real polynomials using quadratic iteration. *SIAM Journal on Numerical Analysis* **7**, 545–566.
